# VMMC Devices—Introducing a New Innovation to a Public Health Intervention

**DOI:** 10.1097/QAI.0000000000000967

**Published:** 2016-05-24

**Authors:** Renee Ridzon, Jason Bailey Reed, Sema K. Sgaier, Catherine Hankins

**Affiliations:** * Department of Epidemiology, Boston University School of Public Health, Boston, MA;; † Jhpiego, An affiliate of Johns Hopkins University, Washington, DC;; ‡ Department of Global Health, University of Washington, Seattle, WA;; § Surgo Foundation, Seattle, WA;; ‖ Department of Global Health and Population, Harvard T. H. Chan School of Public Health, Boston, MA;; ¶ Department of Infectious Disease Epidemiology, Faculty of Epidemiology and Population Health, London School of Hygiene and Tropical Medicine, London, England; and; # Amsterdam Institute for Global Health and Development and Department of Global Health, University of Amsterdam, Amsterdam, the Netherlands.

**Keywords:** voluntary medical male circumcision, circumcision, circumcision devices, innovation, introduction

Innovation, whether related to drugs, vaccines, or even program design, can allow services to be provided in a timely and efficient manner and to be appealing to beneficiaries. Examples such as point-of-care CD4 cell counts, rapid malaria diagnostic tests, tuberculosis diagnosis by cartridge-based nucleic acid amplification test, rapid HIV test kits (and more recently HIV self-testing), and fixed-dose combination antiretroviral therapy innovations in HIV treatment and prevention improve the efficiency and reach of services. Choice is also an important factor in acceptability and uptake of health services, as seen in contraceptive programs where an increased number of options are associated with increased contraceptive uptake.^1^ A recent example of an innovation with potential impact is medical devices developed for adolescent and adult voluntary medical male circumcision (VMMC) HIV prevention programs.

VMMC progress in the 14 priority countries of southern and East Africa is an impressive public health HIV prevention effort. By the end of 2015, 10 million males had been provided with medical circumcision by HIV prevention programs, the vast majority also receiving HIV testing, many for the first time.^2^ Additionally, VMMC was one of 19 global health priorities referred to by the Copenhagen Consensus Centre as a ‘best-buy’ to reach the sustainable development goals.^3^ Beyond the prevention of HIV infection, added benefits of VMMC include prevention of other sexually transmitted infections such as herpes simplex virus and human papillomavirus infections.^4,5^ Not to be forgotten is the fact that an uninfected partner is the best protection against the sexual transmission of HIV, and therefore, the HIV prevention beneficiaries of male circumcision include women and also men. A model for Zambia predicts that reaching a goal of 80% circumcision among males of 15–49 years would reduce HIV incidence among females as much as 26% in the long term.^6^ Models predict that in some countries, almost half of HIV infections averted within a generation's time of VMMC scale-up will be among women.^7^

Achieving VMMC coverage of priority populations is not without challenges and complexities, including cultural barriers, establishment of new supply chains, need for high-quality services, creation of demand for services, funding, and need for efficiencies for service delivery.^8^ Given the goal of VMMC scale-up at a time of decreasing resources for primary prevention modalities, strategies and innovations are needed to improve accessibility, increase uptake of services, ensure sustainability, and improve cost efficiencies, all while maintaining public trust and safety of the program. In contrast to conventional surgical methods, medical devices for circumcision of adults and adolescent males are an innovation that may alleviate some scale-up challenges through the elimination of complexities inherent to surgery, including the need for sutures and injected anesthesia. Device-based techniques are typically shorter, easier to learn, simpler to perform, and have been safely conducted by nurses. Devices have thus been promoted as a means to extend the reach of programs, reduce costs, and potentially increase demand and program efficiency, while maintaining or even improving on safety.^9^ Advantages need to be weighed against the requirement that clients return 7 days after device placement for removal, whereas follow-up after surgical VMMC is voluntary. Two medical devices for adolescent and adult VMMC, PrePex and ShangRing, have been prequalified by World Health Organization (WHO) and can be used in VMMC programs supported by the President's Emergency Plan for AIDS Relief (PEPFAR) and the Global Fund for AIDS, Tuberculosis and Malaria.^10^

Initial research on devices demonstrated safety and acceptability in the setting of clinical trials.^11,12^ Despite this, additional questions that cannot be answered in the clinical trial setting remain, including requirements and timelines for in-country regulatory processes, safety with use in routine service delivery settings (including at fixed, outreach, and mobile service delivery sites), strategies for integration of devices into ongoing programs, client demand, impact on program reach, and the cost of services. To further explore these issues, the Bill & Melinda Gates Foundation and PEPFAR developed a systematic research agenda, independent from device manufacturers, to understand the potential of devices to accelerate VMMC scale-up. As part of the evaluation agenda, it was also important that questions be addressed in contexts as close as possible to “real-life” scaled programs.

This collection of 16 articles examines research on the introduction of the 2 devices prequalified by WHO, PrePex, and ShangRing. These studies provide a firm body of evidence to inform development of recommendations regarding device introduction into VMMC programs already operating at scale. We offer a brief description of the findings on introduction, including on safety, supply, demand, and cost of VMMC using devices, as summarized in Figure 1.

**FIGURE 1.**
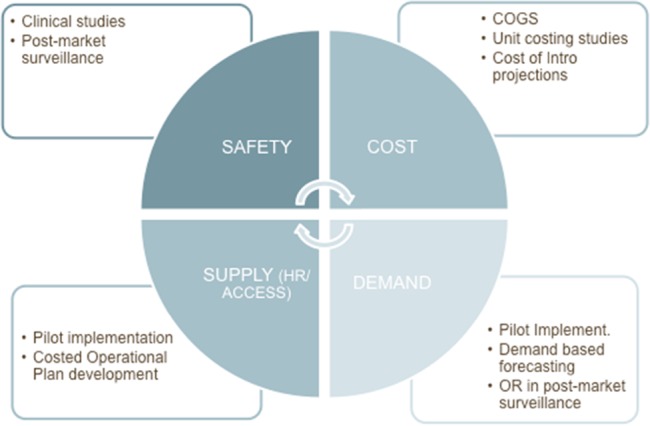
Framework for systematically assessing the role of devices in accelerating the scale-up and improving the efficiency of the VMMC program. COGS, cost of goods sold; HR, human resources; OR, operational research.

## SAFETY

Given that VMMC is an elective prevention intervention, comprised of a medical procedure with a permanent outcome performed on young, healthy persons, safety is of paramount importance. Hence, WHO undertakes prequalification to assure the safety and quality of devices to be used in public health programs in low-resource countries that may have weak, limited, or no regulatory oversight of any medical devices by national authorities. The approach taken by WHO to prequalify devices for adolescent and adult VMMC, and also a framework for expanding their use with postmarketing surveillance, is described. In addition to the prequalification steps, the entire VMMC device clinical safety evaluation pathway suggested by WHO is outlined, including a circumcision device classification scheme.^13^ This work continues to serve as an important foundation to the introduction of VMMC devices.

Other articles in this collection present numerous lessons learned, totaling a bounty of good news (Box 1). Introduction studies of PrePex in adults in Malawi, Zambia, Zimbabwe, Mozambique, South Africa, and Botswana confirm that the device is safe, including in the context of routine service delivery by nurses, and that clients are satisfied and would recommend it to their peers.^14–19^ A study of PrePex in Zimbabwean adolescents aged 13–17 years provides the first safety data in this important younger age group, constituting the largest number of clients who have sought VMMC services thus far.^20^ Although a greater proportion of adolescents versus adults are anatomically ineligible for PrePex, adverse event rates, perceptions of pain, and healing time are all less than observed using PrePex in adults.^20^ Providers quickly become proficient with the use of devices, and adverse event rates with ShangRing are no different with newly trained compared to experienced providers.^21^ A study comparing the safety of using all available Shang-Ring sizes to using fewer sizes shows both schemes to be safe.^22^ This type of operational study could translate into supply chain efficiencies for this device. Good outcomes are also seen with delivery of services in mobile units, which will allow programs to extend reach to more remote areas.^23,24^

BOX 1. **Scope of supplement and lessons learned from studies included in this supplement**

**Scope:**

This supplement contains 16 articles from 8 countries examining different aspects of the introduction of devices into VMMC and focus on the following:

- Safety and acceptability of device introduction in Botswana, South Africa, Malawi, Zambia, Zimbabwe, Mozambique, and Kenya^14–20,22–24^
- The WHO-defined pathway for the evaluation of devices for VMMC programs for HIV prevention^13^
- Estimated unit costs of device-based VMMC and cost of device introduction^27,28^
- Impact of devices on demand for VMMC services^26^
- Provider training and client education.^21,25^

**Lessons learned:**

- Devices are safe and acceptable in routine service delivery
- In the setting of limited capacity for national regulatory oversight, international partners needed to work with WHO to establish a process for assurance of device safety and quality
- Initial implementation of devices has failed to demonstrate potential advantages with regard to efficiency and cost
- Introduction will require planning and ongoing review of operational procedures to resolve challenges that arise
- As greater experience with devices is gained, risk factors for adverse events and delayed healing may be identified, along with risk mitigation, allowing tailored messaging to at-risk clients
- In settings with health care worker shortages, urgent referral of clients in need to skilled surgeons within 6–12 hours may not be possible
- A forecasting study shows that in the context of wide-scale awareness and availability of circumcision services, the PrePex device has potential to improve incremental demand for VMMC
- With regard to the PrePex device:
  - Safety in adolescents has been demonstrated but many adolescents are ineligible
  - Removal pain is experienced by nearly all clients; better control and education about pain is needed
  - Odor is experienced by most clients while the device is in place; expectation setting about this outcome is needed
- With regard to the ShangRing device:
  - Men's understanding and retention of abstinence counseling are good and retained during the healing period
  - Performance of newly trained providers is equal to that of experienced providers
  - Use of a fewer number of sizes is safe and acceptable and could ease supply chain logistics.

There are some important cautionary notes as well. Even severe adverse events nearly always resolve without long-term sequelae; however, in settings where skilled surgeons are mostly located in urban centers, referral of clients who require surgical management of device-related complications within the recommended time frame of 6–12 hours may not be possible.^18^ Although PrePex is safe in adolescents, immature anatomy precludes use in a significant proportion of these younger clients, and other methods will be needed.^20^ Reports of the nearly universal experience of pain at the time of PrePex removal and the development of odor while the device is in place present potential barriers to uptake and point to the need for better pain control with the use of this device and for appropriate client expectation setting concerning odor.^14–17,19^ In response to these findings, studies to optimize pain and odor control are ongoing.

## SUPPLY AND DEMAND

Evaluation of data and operational findings regularly present opportunities for program improvement. During delivery of mobile services in South Africa, periodic assessments allowed improvements in logistics and service delivery.^24^ Analysis of clinical data from fixed and outreach VMMC sites in Kenya permitted identification of risk factors for delayed healing with the potential for delivery of targeted messages for at-risk clients.^22^ Studies on healing with both devices reveal that men retain abstinence messages; study findings can be used to strengthen counseling messages to ensure adherence to instructions and avoidance of complications, such as device displacement/slippage.^17,25^ The impact of devices on demand is analyzed using a market research–based approach to determine the population segments where increases in uptake resulting from introducing device options may be most likely to occur. Data from Zimbabwe and Zambia predict a preference for the PrePex device and substantial incremental demand.^26^

## COST

The 2 PrePex costing studies reveal that the device itself makes up a large part of the procedure unit cost. In modeling studies and simulated analyses, devices have yet to show a significant cost advantage compared with surgery. A costing exercise from Mozambique shows that surgery with reusable instruments is significantly less costly than use of PrePex at current device unit costs, and achievement of cost savings with devices may require novel delivery models.^27^ As the model from Zambia points out, there are costs associated with devices in addition to the basic circumcision unit costs that include training, introduction, and product registration.^28^ Elsewhere, it has been highlighted that low demand and underutilization of VMMC services are the largest drivers of high unit costs.^29^ Although market research–based methods forecast increased demand because of availability of devices, the actual effect on demand, and in turn costs, in real-world settings is yet to be seen.

## CONCLUSIONS

This collection of articles presents new research that underscores the challenges and opportunities for the use of medical devices in VMMC programs, as an alternative to conventional surgical approaches. Findings highlighted in this supplement will be important as the introduction of VMMC devices continues. Key questions posed by decision makers—will the innovation be more attractive than the existing options, extend the reach of programs, be more cost effective, be as safe as the current standard of care?—are not answered simultaneously. As demonstrated through these articles, answers are revealed across a series of activities, building step-wise on results from previous work. Solutions to some of the most critical questions are not knowable until substantial investments of time, effort, and money have been committed. For example, real-world acceptability may not be fully known until new innovations or products are truly widely available as a routine service option. Likewise, the most consequential adverse events may be exceptionally rare and not appreciated until after large numbers of clients have been reached. Readers are encouraged to consider nuanced aspects of product introduction, including applicability for specific populations and settings, potential program efficiencies, and possible impact on male circumcision unit costs.

Information presented here covers some of the operational aspects of device introduction. The need for pilot studies, objective data on safety, examination of acceptability and costs, and documentation of the challenges encountered is not unique to this innovation. We hope that this example can serve as a case study for the introduction of other health products for large scale HIV prevention and treatment.
